# Role of Mitophagy in Regulating Intestinal Oxidative Damage

**DOI:** 10.3390/antiox12020480

**Published:** 2023-02-14

**Authors:** Xiaobin Wen, Lixin Tang, Ruqing Zhong, Lei Liu, Liang Chen, Hongfu Zhang

**Affiliations:** 1 State Key Laboratory of Animal Nutrition, Institute of Animal Science, Chinese Academy of Agricultural Sciences, Beijing 100193, China; 2 State Key Laboratory for Molecular Biology of Special Economic Animals, Institute of Special Animal and Plant Sciences, Chinese Academy of Agricultural Sciences, Changchun 130112, China

**Keywords:** ROS, oxidative stress, mitophagy, PINK1-Parkin, intestinal damage

## Abstract

The mitochondrion is also a major site for maintaining redox homeostasis between reactive oxygen species (ROS) generation and scavenging. The quantity, quality, and functional integrity of mitochondria are crucial for regulating intracellular homeostasis and maintaining the normal physiological function of cells. The role of oxidative stress in human disease is well established, particularly in inflammatory bowel disease and gastrointestinal mucosal diseases. Oxidative stress could result from an imbalance between ROS and the antioxidative system. Mitochondria are both the main sites of production and the main target of ROS. It is a vicious cycle in which initial ROS-induced mitochondrial damage enhanced ROS production that, in turn, leads to further mitochondrial damage and eventually massive intestinal cell death. Oxidative damage can be significantly mitigated by mitophagy, which clears damaged mitochondria. In this review, we aimed to review the molecular mechanisms involved in the regulation of mitophagy and oxidative stress and their relationship in some intestinal diseases. We believe the reviews can provide new ideas and a scientific basis for researching antioxidants and preventing diseases related to oxidative damage.

## 1. Introduction

Under the influence of various external and internal causes (such as harmful substances and inflammation), the body produces excessive ROS, which is in an imbalance with the antioxidant defense, leading to oxidative stress and subsequent intestinal cell apoptosis and tissue damage [1,2,3]. Some diseases related to cell death, including neurodegenerative diseases, tumors, and inflammation, are related to oxidative stress. Inflammatory bowel disease (IBD) is a group of chronic relapsing-remitting inflammatory conditions, comprising two main subtypes: Crohn’s disease (CD) and ulcerative colitis (UC). IBD has emerged as a growing problem in the world, and affects close to seven million individuals, with this number expected to rise in the next decade [4,5]. However, the exact mechanism of IBD remains unclear. The search for novel IBD therapeutic compounds and the development of IBD therapeutic strategies are ongoing challenges. A growing number of studies have found that ROS-induced oxidative stress is an important factor in the development of IBD. Oxidative stress leads to intestinal barrier damage and bacterial invasion, which in turn stimulates the immune response and induces IBD [6].

Currently, damage mechanisms and prevention of oxidative stress are fields of intense interest. Mitochondria are vital energy-generating organelles in eukaryotic cells, and it is the most important precondition for normal cellular function to maintain the structural and functional integrity of mitochondria [7,8]. In addition to producing cellular energy, causing free radicals to form, and initiating apoptosis, mitochondria are the major source of ROS within cells and are the primary target for them [9,10]. ROS can damage the mitochondria and further decrease the efficiency of the mitochondrial electron transfer chain (ETC), resulting in a positive feedback loop of mitochondrial ROS generation and mitochondrial oxidative damage [11,12]. Mitophagy is a protective mechanism to reduce oxidative stress on cells discovered in recent years. Studies have shown that ROS can induce mitophagy by activating the PINK1–Parkin pathway, and clear damaged and redundant mitochondria, playing a key regulatory role in maintaining mitochondria health and preventing diseases related to oxidative damage [13,14,15]. By contrast, ROS scavengers block the autophagosome formation and the subsequent degradation of engulfed proteins [16]. Mitophagy selectively removes damaged, aging, and ROS-overproduced mitochondria through autophagy, promotes mitochondrial regeneration and recycling, and maintains normal cellular function [17,18,19]. It follows that there is a strong link between oxidative stress and mitophagy. Thus, the selective degradation of mitochondria by autophagy (mitophagy) is important for cellular homeostasis and disease prevention [20]. This paper reviews the role of mitophagy in alleviating oxidative damage. The major findings linking mitophagy, oxidative stress, and intestinal diseases such as CD and UC, are discussed in this review.

## 2. Oxidative Stress and Intestinal Oxidative Damage

Oxidative stress, a concept of pathology that was first proposed by Sohal in 1990 [21] refers to disorders of the oxidation system and antioxidant system caused by increased free radicals or (and) decreased scavenging capacity, leading to an oxidative damage process caused by the accumulation of free radicals in the body. Generally, oxidative stress is caused by the excessive production of ROS. ROS are oxygen-containing chemical species that are highly reactive, such as hydrogen peroxide and superoxide anion, mainly produced by oxidative phosphorylation (OXPHOS) in mitochondria [22,23]. Within physiological levels, ROS can function as signaling molecules that contribute to cellular growth and development, metabolic regulation, and immune and other biological processes [24]. In addition, the endogenous antioxidant system will promptly remove ROS, thereby maintaining the balance of the oxidation-reduction (redox) status of the body [25]. However, antioxidant defenses cannot counteract excessive ROS production when the body has been stimulated by stressors or infected with pathogenic bacteria [26]. As a result, excess ROS causes cellular oxidative damage, such as lipid peroxidation, protein oxidation, and DNA damage. To prevent further oxidative damage, injured cells activate a series of defensive responses, such as increasing antioxidant enzyme activity and initiating lysosomal degradation pathways.

### Intestinal Oxidative Damage

Oxidative stress induces human nervous, immune, cardiovascular, and cerebrovascular diseases and endangers animal growth, milk production, reproduction, and disease resistance, causing huge economic losses for animal husbandry [27,28]. Oxidative stress is associated with weaning stress, which has significant effects on the health and function of the intestine. In animal production, early weaning induces excessive ROS production in piglets, which is one of the important reasons for the induction of “piglets weaning stress syndrome.” Energy is essential for the renewal and normal function of intestinal epithelial cells, and its production is accompanied by the production of ROS in the oxidative respiratory chain of mitochondria [29]. Therefore, the intestine is “the central organ for stress.” As the intestine of weaned piglets has not yet fully developed and matured, their anti-stress ability is weak. When subjected to oxidative stress, the gastrointestinal tract is easily damaged. Despite the protective barrier provided by the epithelial layer, ingested substances and pathogens can further contribute to oxidative stress by activating epithelial cells, polymorphonuclear neutrophils causing inflammation, and macrophages producing inflammatory cytokines and other mediators.

The intestine is the leading site of nutrient absorption, and villi health impacts nutrient absorption vastly [30]. The intestinal epithelial cells continuously undergo self-renewal and completely regenerate every few days [31]. Regenerated intestinal epithelial cells are particularly sensitive to free radicals [32]. As an example, ROS can peroxide unsaturated fatty acids in phospholipids of cellular membranes, resulting in impaired membrane function [33]. Lipid peroxidation from free radicals leads to structural and functional changes in gastrointestinal epithelial cells. Moreover, during oxidative stress situations, cell cycle lengths of intestinal epithelial cells became longer and the villus crypt dysgenesis hinders nutrient absorption [34]. All the above results in a decrease in animal performance and an increase in mortality.

Intestinal tight junction proteins are pivotal components of the intestinal mucosal barrier, and play an important role in maintaining the stability of the intestinal epithelial barrier and permeability, which can prevent toxins and pathogens from entering the blood from the intestine [35,36]. During oxidative stress, free radicals change the structure of tight junction proteins, resulting in intestinal barrier dysfunction and putting animals at risk of various infections. Previous studies found that oxidative stress destroys the tight junction proteins, which in turn leads to the passive diffusion of water and ions from the intestinal mucosa into the lumen and further development into diarrhea [37,38]. Oxidative stress induces a significant decrease in TEER and permeability, as well as a significant decrease in the abundance of the tight junction proteins claudin-1 and ZO-1 in IPEC-1 cells [39]. At present, it is believed that oxidative stress mainly regulates the tight junction by phosphorylating related proteins, or destroys the tight junction by improving the activity of metalloproteinases and promoting the expression of inflammatory factors. Elisa et al. reported that free radical phosphorylated Tyr-398 and Tyr-402 in Occludin, thereby preventing its interaction with ZO-1 and destabilizing its assembly at the tight junctions [40]. This was also echoed by similar findings from other studies. ROS phosphorylated ZO-1 and caused its degradation, leading to the separation of Claudin-4 and Occludin from the intestinal epithelial tight junction complex [41]. High concentrations of free radicals and ROS seem to activate matrix metalloproteinases (MMPs), and activate MMPs then mediate the degradation of the tight junction proteins [42,43]. In addition, free radicals could also activate small G-protein (RhoA) by promoting the secretion of INF-γ, which leads to the disintegration of the intercellular tight junction complex by up-regulating Rho kinase (ROCK) factor [44].

In the intestinal tract, beneficial and potentially harmful bacteria are in a dynamic balance [45]. As mentioned above, oxidative stress increases intestinal permeability, which in turn leads to the invasion of a large number of harmful substances, competitively inhibits the colonization of the commensal bacteria, and leads to microflora disorder. When oxidative stress occurs, the intestinal epithelium passively diffuses oxidation products, thus increasing the oxidation potential and stimulating the growth of aerobic bacteria [46]. Moreover, ROS can stimulate the production of IL-6 and other inflammatory factors through the NF-κB pathway, increasing facultative anaerobic bacteria and exacerbating intestinal dysbiosis [47,48]. The effect of intestinal microflora on intestinal redox balance is complex. Both beneficial and pathogenic bacteria stimulate the production of free radicals. Some beneficial bacteria can reduce the concentration of free radicals by producing metabolites such as antioxidant enzymes or SCFAs, such as *Bifidobacterium* [49] and *Lactobacillus plantarum* FC255 [50]. The antioxidant properties of probiotic bacteria have been elaborated on in previous studies [51]. Fecal microbiota transplantation can alleviate NEC-induced intestinal and systemic inflammatory responses and alleviate intestinal oxidative damage [52]. When certain Gram-negative bacteria such as E. coli die, parts of their cell walls become powerful endotoxins that cause intestinal inflammation and oxidative stress and are also present in other organs such as the liver and brain. In summary, when oxidative stress occurs in the body, timely regulation of intestinal flora may be one of the effective ways to enhance oxidative stress and further improve intestinal injury.

## 3. Mitophagy Pathway

**Figure 1:**
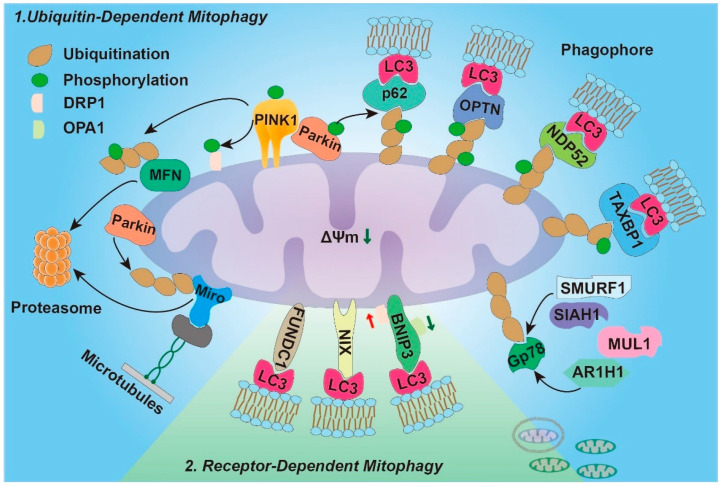
Molecular mechanism of mitophagy. In the PINK1–Parkin pathway, decreased MMP leads to the accumulation of PINK1 to the OMM, promoting Parkin recruitment. Parkin ubiquitinates several outer membrane components, and PINK1 then phosphorylates the poly-Ub chains. The poly-Ub chains on the OMM allow the interaction of mitochondria with LC3 for autophagic degradation through specific adaptors, such as p62, OPTN, TAX1BP1, and NDP52. Gp78, SMURF1, MUL1, SIAH1 and ARIH1 represent alternative E3 ubiquitin ligases targeting OMM proteins before mitophagy. Mitochondrial dynamics and motility are modulated by the PINK1–Parkin pathway by targeting MFN, DRP1 and Miro for degradation by proteasomes. In receptor-mediated mitophagy, BNIP3, NIX and FUNDC1 mitophagy receptors mediate mitochondrial elimination indirectly by interacting with LC3.

Not only do mitochondria produce ROS in large quantities, but they are also a primary target for ROS attacks [53,54]. Physiological ROS is important for cellular function and survival signaling, yet excess ROS-elicited oxidative stress leads to cell death [55,56]. There is a vicious cycle in which initial mitochondrial damage leads to ROS production, which creates further mitochondrial damage and eventually leads to massive intestinal cell death. To protect cells from oxidative damage, cells have developed an elaborate self-protective mechanism for selective degradation of the dysfunctional mitochondrion before it hurts the cell, called mitochondrial autophagy, or mitophagy [57]. Mitophagy is a term that was introduced by Lemasters in 2005 and designates the removal of dysfunctional mitochondria and their harmful byproducts and oxidative species to help maintain homeostasis [58,59]. Specifically, under the stimulation of starvation, hypoxia, energy deficiency, hormones, and exogenous infection, the bilayer membrane structures recognize and specifically wrap the mitochondria that are depolarized, dysfunctional, or produce high levels of ROS, and fuse with lysosomes to degrade these mitochondria, reducing the release of ROS and some pro-apoptotic factors from mitochondria. In mitophagy, the regulatory pathways can be classified as ubiquitin-dependent or ubiquitin-independent (receptor-dependent), respectively, based on whether the ubiquitin of the receptor recruit is required or not [60,61] (Figure 1).

### 3.1. Ubiquitin-Dependent Mitophagy

PTEN-induced putative kinase 1 (PINK1)-cytosolic E3 ubiquitin ligase Parkin pathway represents one of the most studied ubiquitin-dependent mechanisms of mitophagy [59,62]. PINK1–Parkin has multiple roles in mitochondrial physiology, including mitochondrial dynamics, biogenesis, transport, and recruitment of autophagic machinery, to ensure the elimination of defective organelles [61]. PINK1 is an outer mitochondrial membrane (OMM) protein with Ser/Thr protein kinase activity [59]. Parkin (*PARK2*) is an E3 ubiquitin-protein ligase that mediates protein degradation and signal transduction in the cell [63]. In mammals, PINK1 and Parkin are expressed in many organs and tissues, such as the brain, skeletal muscles, heart, gut, and liver. In the inner mitochondrial membrane (IMM), PINK1 is continuously imported through translocase complexes between the outer and inner membranes, and is then degraded by various proteases, such as mitochondria-processing protease (MPP) and the inner membrane presenilin-related rhomboid-like protease (PARL) [62,64]. As a result, PINK1 is maintained at a low level in normal mitochondria, keeping its autophagy capacity at normal levels. When mitochondria are damaged or the membrane potential (ΔΨm) is depolarized, the inward transport and degradation of PINK1 will be inhibited, resulting in the accumulation of PINK1 in the OMM. This means that PINK1 likely acts as a mitochondrial damage sensor, activating Parkin to translocate from the cytoplasm to mitochondria [59]. In addition, the disruption of PINK1 phosphorylation blocks the Parkin’s translocation, while the deficiency of Parkin phosphorylation abolishes PINK1–Parkin-induced cell death [65]. As a consequence of its translocation, PINK1-dependent phosphorylation alters Parkin conformation, causing it to bind mitochondrial surfaces and trigger E3 ligase activity [61,66,67]. PINK1 also phosphorylates ubiquitin (Ub) and poly-Ub chains on dysfunctional mitochondria [61]. The inactivated Parkin binds to phospho-Ub and promotes its activation by PINK1. Parkin mediates the feedforward mechanism to generate poly-Ub chains, which is the substrate of PINK1, thus amplifying the autophagy signals [61,66,68]. Therefore, the significance of PINK1 in the mitochondria is needed in cell-protective characteristics for combating oxidative stress [69]. As a result of poly-Ub chains being phosphorylated by PINK1 and serving as an “eat me” signal for the autophagic machinery, the adaptor proteins recognize phosphorylated poly-Ub chains on mitochondrial proteins (p62, OPTN, NDP52, TAXBP1) and bind with the LC3 receptor to begin autophagosome formation [61].

In addition to mitophagy, the PINK1–Parkin pathway plays a vital role in mitochondrial movement as well [70,71]. Mitochondrial fission and fusion are affected at different levels by the PINK1–Parkin pathway [72]. Mitochondrial fusion is induced by the homo- or hetero-dimerization of the mitofusins (MFNs) 1 and 2, which tether the mitochondria together, pull the mitochondria that are in close proximity, and mediate fusion [73,74]. On the other hand, the mitochondrial fission process is orchestrated by the GTPase dynamin-related protein 1 (DRP1) [72]. DRP1 activity is indirectly triggered by PINK1 and promotes the fission of dysfunctional mitochondria, which enables their autophagic degradation [72,75]. In coordination with DRP1 activation, PINK1–Parkin-mediated phosphorylation and proteasomal degradation of MFN1 also contribute to mitochondrial fragmentation and the removal of dysfunctional organelles [76]. Additionally, MFN2 may function as a scaffold protein to facilitate Parkin translocation when mitochondrial damage occurs [61]. Specifically, MFN2 mediates the recruitment of Parkin to damaged mitochondria, and PINK1 phosphorylates MFN2 and promotes its ubiquitination by Parkin [62,76]. In this way, PINK1–Parkin activation causes rapid ubiquitination and degradation, preventing damaged mitochondria from fusing with healthy organelles [62,76]. There is evidence that healthy organelles and mitochondria contacts play a critical role in mitophagy, where PINK1–Parkin activation and deubiquitylation events occur. In addition to this, mitochondrial membrane potential dissipation stimulates PINK1-dependent phosphorylation of Miro, a Rho–GTPase of the OMM that anchors mitochondria to the cytoskeleton [61]. By ubiquitinating Miro, Parkin promotes its degradation while inhibiting mitochondrial transport. This causes mitochondria to dissociate from tubulin and separate from the mitochondrial network [61,70]. Therefore, blocking mitochondrial transport and enhancing fission could facilitate mitophagy, possibly by reducing mitochondria’s mobility and enabling them to be sequestered by autophagosomes. In addition, Parkin can also interact with Ambra1 to activate class III PI3K complexes (Vps34) around mitochondria to promote mitochondrial autophagy [77,78].

In addition to Parkin, a number of other ubiquitin E3 ligases are involved in the regulation of mitophagy, including Gp78, SMURF1, SIAH1, MUL1, and ARIH1 [61,62,79,80,81]. Their presence on the mitochondrial surface triggers the recruitment of autophagy adaptors, including optineurin (OPTN), nuclear dot protein 52 (NDP52), and p62, among others, by generating ubiquitin chains. In autophagy, autophagic components, like unc-51-like autophagy activating kinase 1 (ULK1), double FYVE-domain-containing protein 1 (DFCP1), and WD repeat domain, phosphoinositide interacting 1 (WIPI1), are recruited for the biogenesis of phagophores and expansion of autophagosome membranes [61,79,82].

### 3.2. Ubiquitin-Independent Mitophagy (Receptor-Dependent)

Apart from ubiquitin-dependent mitophagy, mitochondrial proteins serve as mitophagy receptors, mediating ubiquitin-independent mitophagy, also known as receptor-dependent mitophagy [61,81]. The OMM proteins BNIP3 (BCL2 interacting protein 3), NIX (NIP3-like protein X), and FUNDC1 (FUN14 domain-containing protein 1) are mitophagy receptors that fine-tune mitochondrial populations in response to various stimuli [61]. These mitophagy receptors directly or indirectly interact with the LC3 protein and lead to the selective degradation of mitochondria [83]. In addition, the activities of these mitophagy receptors are regulated by the phosphorylation status of their LIR [84]. During hypoxic conditions, BNIP3 and NIX, which belongs to the subfamily of BH3-only proteins, are induced and localized to the OMM through its carboxy-terminal transmembrane domain [83]. BNIP3 and NIX share the same LIR domains, and these LIR domains help BNIP3 and NIX bind to LC3 on autophagosomes. BNIP3 interferes with mitochondrial dynamics, promoting the fission of damaged organelles through the disassembly and release of OPA1, and recruitment of DRP1 to the mitochondrial surface [85,86]. In addition, BNIP3 increases mitophagy by suppressing the cleavage of PINK1. BNIP3 and its homolog NIX can not only promote apoptosis through the BH3 domains, but also induce mitophagy through the LIR motifs [87]. During reticulocyte development, NIX was shown to be required for mitochondrial clearance, while BNIP3 was identified to mediate mitophagy in cardiomyocytes and liver cells [85,86]. In addition to participating in ubiquitin-independent mitophagy, NIX also acts as a substrate of Parkin, recruiting NBR1 and ultimately triggering mitophagy [62]. FUNDC1 is located in the OMM and is associated with mitophagy under hypoxia or mitochondrial membrane potential dissipation [83]. FUNDC1 binds to LC3 via a conserved LIR motif and this interaction is enhanced under hypoxic conditions, facilitating mitochondrial engulfment by autophagosomes [72]. Phosphorylation of FUNDC1 and the consequent inhibition of FUNCD1-mediated mitophagy leads to excessive ROS accumulation [72,88]. Knockout of endogenous FUNDC1 significantly inhibited hypoxia-induced mitophagy [89]. It is attractive to hypothesize that NIX, BNIP3, and FUNDC1 act synergistically during hypoxia, with the results that FUNDC1 selectively targets mitochondria to autophagosomes formation via NIX- and BNIP3-mediated Beclin 1 activation [70]. Apart from the above, other mitophagy protein receptors of OMM include autophagy and Beclin 1 regulator 1 (AMBRA1), FKBP prolyl isomerase 8 (FKBP8), BCL2 like 13 (BCL2L13), and Prohibitins (PHB) [62,90].

## 4. Mitophagy and Oxidative Stress in Intestinal Disease

ROS regulates mitophagy by mediating the PINK1–Parkin pathway through its receptors or effector proteins. PINK1–Parkin pathway regulation of mitophagy also plays an important role in different oxidative damage diseases. The continuous differentiation and renewal of intestinal cells require a large amount of energy, which is accompanied by the production of ROS in the oxidative respiration chain of mitochondria [72,91]. Therefore, ROS is extremely active in intestinal epithelial cells, and its excessive accumulation can easily induce oxidative damage and lead to mitophagy, which is an important factor in the occurrence of various intestinal diseases.

Ulcerative colitis (UC) is a chronic inflammatory bowel disease with inflammation and ulceration of the colonic mucosa [92]. Oxidative stress and mitochondrial dysfunction are related to the pathogenesis of UC, and the incidence and severity of UC are correlated with the increase in ROS. Studies have found that high concentrations of oxidative molecules can be detected in plasma, serum, and even exhaled gas and saliva of UC patients, and the severity of UC is positively correlated with oxidative stress [93]. It has been confirmed that MnSOD and CAT expressions are decreased in UC, while MDA and GPX2 expressions are increased [94,95,96]. The antioxidant appearances were the same in the DSS-induced colitis mouse model, and the expression levels of ROS, IL-1β, and IL-18 in mitochondria decreased after treatment with the mitochondrial-targeted antioxidant MitQ [97]. Therefore, oxidative stress plays a critical role in UC pathogenesis and can be used as a therapeutic target and evaluation indicator. DSS-induced mitochondria in the rat model were in ridge collapse, expansion, and fragmentation states, and the membrane potential changes were reduced by 70% as compared with those in the normal mice [98]. Oxidative stress results in mitochondrial damage in UC patients, PINK1 and Parkin expressions are increased in UC, and mitophagy is activated as a protective mechanism [99]. Mitochondria can regulate the pro-inflammatory response of cells by activating molecular complexes of inflammasomes. ROS can cause NLRP3 to form a complex with apoptosis-associated speck-like protein that further enforces the precursor Caspase-1 to form the active NLRP3 inflammasome, which stimulates caspase-1 cleavage and releases activated IL-1β and IL-18 [100]. Mitochondria can also induce intestinal inflammation by signal transduction through pattern recognition receptors [101]. In conclusion, in ulcerative colitis, cells clear damaged mitochondria and overloaded ROS through mitophagy, thus protecting the colonic mucosa from damage. However, the exact route is still unclear, and further research is required in the future.

Crohn’s disease (CD) is referred to as regional enteritis due to the typical pattern of multifocal involvement with intervening normal tissue, “skip lesions”, which can involve any area of the gastrointestinal tract [102]. In patients with CD, oxidative stress and the accumulation of damaged intracellular macromolecules are important contributors to mucosal deterioration and increased intestinal permeability [103,104,105,106]. The ROS levels in CD patients are imbalanced with the antioxidant defenses. Moreover, the impaired and reduced Paneth cell is an important mechanism for the development of intestinal pathology in CD. Khaloian et al. reported that CD-like inflammation was negatively correlated with reduced Paneth cell function and LGR5-expression, and mitochondrial metabolic disorders can be induced by the absence of HSP60 in intestinal stem cells [107]. Jackson et al. also identified Paneth cells as highly susceptible to mitochondrial dysfunction and play a key role in ileitis pathogenesis, with translational implications for the subset of Crohn’s disease patients exhibiting Paneth cell defects [108]. Mitochondrial protein abundance in the ileum and colon of CD patients was significantly lower than that in the normal population, and CD patients show high mitochondrial activity of electrical signals in the ileum [109,110]. OPTN is a primary receptor required for the selective autophagy of damaged mitochondria [111]. Studies have found that OPTN is closely related to CD [112]. At present, there are few studies on CD and mitophagy. However, autophagy-related gene polymorphism is indeed associated with the pathogenesis of CD.

While the role of mitophagy in the development of IBD is now beginning to be understood, there is still a lot to be discovered. Additionally, some researchers have conducted many experiments using a model simulating intestinal oxidative damage. H_2_O_2_ is both an exogenous and endogenous pro-oxidative mediator that can lead to lipid peroxidation within the cell membranes and destroy the structure and function of the cell membrane, so it has been utilized in establishing an oxidative stress model in vitro [113,114,115,116,117]. A recent study reported that curcumin can effectively ameliorate H_2_O_2_-induced oxidative stress, intestinal epithelial barrier injury, and mitochondrial damage in IPEC-J2 cells in a PINK1–Parkin mitophagy-dependent way, and the authors also found depletion of Parkin abolished curcumin’s protective action on anti-oxidative stress [118]. Moreover, the same results were obtained with other drugs, such as sodium butyrate [119], selenium nanoparticles (SeNPs) [120], and resveratrol [121]. Consistent with in vitro data, dietary curcumin protected intestinal barrier function, improved redox status, alleviated mitochondrial damage, and triggered mitophagy in a well-established pig oxidative stress model by challenging with diquat [118,122,123]. Similarly, resveratrol can induce mitophagy in the intestine to alleviate the intestinal barrier dysfunction induced by oxidative stress [122]. From this, we can suggest that the addition of exogenous drugs could regulate mitophagy, thereby attenuating oxidative stress. Therefore, the interplay of the intestinal barrier, mitochondrial, and mitophagy provides insight into the development of therapeutic strategies for the prevention and treatment of intestinal oxidative stress. Moreover, several synthetic and natural chemical compounds have been shown to modulate mitophagy in other common human diseases (Table 1).

## 5. Conclusions

**Figure 2:**
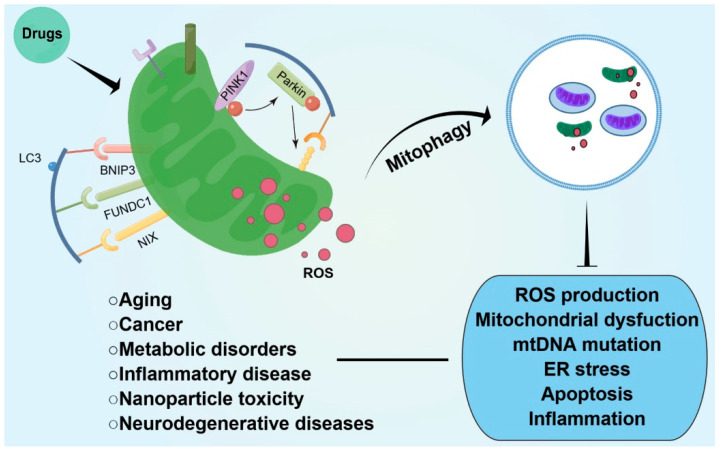
Administration of drugs, which regulate mitochondrial ROS production and mitophagy, improves organismal homeostasis and various disease conditions.

When humans and animals are subjected to oxidative stress, excessive ROS leads to mitochondrial structure and function disorders, further inducing systemic oxidative damage and activating mitophagy. At present, the process and molecular mechanism of mitophagy have been preliminarily clarified, and important research results have been achieved in the removal of damaged mitochondria and other organelles, and their participation in the repair of oxidative damage. However, the causal relationship between mitochondrial dysfunction and disease remains controversial and requires further study. In the future, with further research on the mechanism of mitophagy, we expect to enhance the adaptability to oxidative stress by regulating mitophagy. Meanwhile, finding targeted drugs that can regulate mitochondrial ROS production and mitophagy can be a potential research direction for the prevention and treatment of intestinal oxidative stress. These may also, of course, apply to the study of other common diseases, such as neurodegenerative diseases, tumors, and inflammation (Figure 2).

## Figures and Tables

**Table 1:** Effect and mechanism of the candidate drugs in human diseases.

Drugs	Disease	Model	Effect and Mechanism
Urolithin A	Alzheimer’s disease	human neuronal SH-SY5Y cells; AD mice	Urolithin A induces neuronal mitophagy by increasing key mitophagy-related proteins. It can also enhance the phagocytic efficiency of microglia, and mitigate NLRP3/caspase-1-dependent neuroinflammation [124]
Rapamycin	Parkinson’s disease	SH-SY5Y cells treated with rotenone	Rapamycin enhances mitophagy through inhibition of mTOR to clear the Cytochrome *c*, thereby inhibiting the occurrence of apoptosis caused by rotenone [125]
Pifithrin-α	Diabetes	streptozotocin-treated and *db/db* mice	Pifithrin-α induces mitophagy by promoting Parkin activity through p53 downregulation, then ameliorates mitochondrial dysfunction and glucose intolerance [126]
Resveratrol	Diabetic cardiomyopathy	rats by a high-fat diet combined with STZ injection	Resveratrol alleviates cardiac dysfunction in diabetes by improving mitochondrial function via SIRT1-mediated PGC-1α deacetylation [127]
Rapamycin	Leigh syndrome	Ndufs4^−/−^ mice	Rapamycin delays the onset of neurological symptoms, reduces neuroinflammation, and prevents brain lesions [128]
Ginsenoside Rg3	Hepatitis C Virus	HCV-infected Huh7 and Huh7.5.1 cells	Ginsenoside Rg3 restores the HCV-induced dynamin-related protein 1-mediated aberrant mitochondrial fission process, thereby resulting in the suppression of persistent HCV infection [129]
Panax notoginseng saponins	Hypoxia/reoxygenation	H9c2 cells with H/R injury	Panax notoginseng saponins reduce H/R injury in cardiomyocytes by activating HIF-1α/BNIP3 mitochondrial autophagy signaling pathways [130]
Empagliflozin	Cardiac microvascular ischemia/reperfusion	myocardial ischemia (45 min)/reperfusion (2 h) injury mice	Empagliflozin normalizes mitochondrial fission and fusion, neutralizes supraphysiologic ROS concentrations, and suppresses mitochondrial apoptosis by activating FUNDC1-dependent mitophagy through the AMPKα1/ULK1 pathway [131]
Spermidine	Aging	yeast, flies, nematodes, and human immune cells	Spermidine plays an important role in mitophagy-mediated cytoprotective and anti-aging effects through energy metabolism restoration [132]
